# Ocular Therapeutics and Molecular Delivery Strategies for Neovascular Age-Related Macular Degeneration (nAMD)

**DOI:** 10.3390/ijms221910594

**Published:** 2021-09-30

**Authors:** Aira Sarkar, Vijayabhaskarreddy Junnuthula, Sathish Dyawanapelly

**Affiliations:** 1 Chemical and Biomolecular Engineering, Johns Hopkins University, Baltimore, MD 21218, USA; asarka11@jhu.edu; 2 Drug Research Program, Faculty of Pharmacy, University of Helsinki, Viikinkaari 5, 00790 Helsinki, Finland; 3 Department of Pharmaceutical Sciences & Technology, Institute of Chemical Technology, Nathalal Parekh Marg, Mumbai 400019, India

**Keywords:** ocular therapeutics, age-related macular degeneration, gene therapy, cell therapy, bispecific antibodies, port delivery system

## Abstract

Age-related macular degeneration (AMD) is the leading cause of vision loss in geriatric population. Intravitreal (IVT) injections are popular clinical option. Biologics and small molecules offer efficacy but relatively shorter half-life after intravitreal injections. To address these challenges, numerous technologies and therapies are under development. Most of these strategies aim to reduce the frequency of injections, thereby increasing patient compliance and reducing patient-associated burden. Unlike IVT frequent injections, molecular therapies such as cell therapy and gene therapy offer restoration ability hence gained a lot of traction. The recent approval of ocular gene therapy for inherited disease offers new hope in this direction. However, until such breakthrough therapies are available to the majority of patients, antibody therapeutics will be on the shelf, continuing to provide therapeutic benefits. The present review aims to highlight the status of pre-clinical and clinical studies of neovascular AMD treatment modalities including Anti-VEGF therapy, upcoming bispecific antibodies, small molecules, port delivery systems, photodynamic therapy, radiation therapy, gene therapy, cell therapy, and combination therapies.

## 1. Introduction

Age-related macular degeneration (AMD) is the leading cause of vision loss in developed countries with prevalence rates ranging from 5–40% based on ethnicity [1,2]. In 2020, around 200 million people were suffering from AMD, and projections are nearly 300 million in 2040 worldwide [1,2]. The pathophysiology of AMD includes inflammation mechanisms affecting the retina coupled with oxidative stress. The disease is broadly classified into dry AMD and wet AMD. Dry AMD could progress into wet AMD (neovascular AMD) after the progression of new blood vessels into the retina and subretinal space. Subsequently, these vessels cause bleeding, leakage of serum and fluid retention, distortion in vision, and central vision loss after progression [3]. Various treatment modalities are under consideration, intravitreal biologics (Bevacizumab, Ranibizumab, and Aflibercept) being the standard treatment of care given as intravitreal injections. The intravitreal injection (IVT) route is currently in use for anti-VEGF (Vascular Endothelial Growth Factor) agents. It is an invasive route and causes difficulty to patients, but alternative clinical outcomes are bleak at present [4,5]. Various administrative routes are explored, such as periocular, suprachoroidal, sub-retinal, systemic, and topical routes for the delivery of small molecules, biologics, and gene therapy [6]. Molecular therapies such as cell therapy and gene therapy are in focus due to their restoration ability [7,8,9,10]. AMD is a disease of the posterior eye segment which brings various challenges. There are two basic ways to improve the therapeutic outcome: prolonged delivery of intravitreal drugs and use of various other routes [5]. There were numerous attempts to deliver drugs and biologics from the topical routes due to its simple and non-invasive administration. However, the barriers in delivering biologics to the cornea (Absorption, Distribution, Metabolism, and Excretion (ADME); degradation of biologics; and their interactions with corneal layers) currently prevent the exploitation this route [11]. In general, biologics have longer half-lives in vitreous, and small molecules have shorter half-lives. If the patient misses the clinician’s appointment for any reason, the IVT therapy could decrease the efficacy, hence the need for long-acting injectables or implants. In recent years, nanoparticles (NPs) have been extensively investigated and used in drug delivery and biomedical application [12,13,14,15]. Several delivery vehicles are reported in the literature including, micelles, nanoparticles, polymersomes, liposomes, and reviews on the details of nanoparticle preparation and characterization, including their evaluation with respect to storage stability, toxicity potential, and in vivo fate [16,17,18,19,20,21]. Recently, we have reported a comprehensive review on “Nanodiagnostics and Nanotherapeutics for Age-related macular degeneration” [22] and hence this will not be discussed here. In the present review, we highlight preclinical and clinical studies of wet AMD treatment modalities such as anti-VEGF therapy, including antibodies, bispecific antibodies, small molecules, photodynamic therapy, radiation therapy, gene therapy, and cell therapy. We are also providing information on sustained-release strategies and pharmacokinetics aspects to reduce the need for frequent injections [23].

## 2. Pathogenesis of AMD

**Figure 1:**
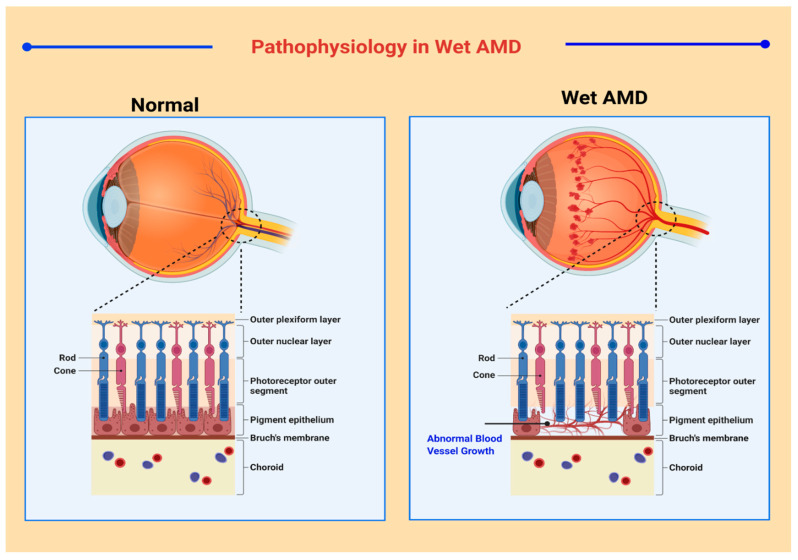
Anatomical and Pathophysiological features of retina cell layers in neovascular AMD. Normal Vision and wet AMD.

A broad spectrum of classification models has been developed for medical and research purposes to distinguish between the various manifestations of AMD. However, there exists no specific classification system that is accepted worldwide [24]. According to one convention, macular degeneration can be broadly classified as wet AMD and dry AMD [25]. Wet AMD, or neovascular AMD (nAMD), is an exudative degeneration that is caused by hyper-expression of VEGF along with rapid and progressive angiogenesis [26]. Pathophysiological features in neovascular AMD are shown in Figure 1. Dry AMD is a non-exudative degeneration that is defined as the accumulation of “drusen” underneath the maculae [27]. Among the cases diagnosed, ~90% of the occurrences are associated to dry AMD whereas ~10% of cases are due to nAMD, mostly as all early forms of AMD are considered as dry AMD. Studies have depicted that 10–15% of non-neovascular AMD cases may progress into wet AMD [28,29].

The Wisconsin age-related maculopathy (ARM) [30] system, a convention based on the photographic retinal grading approach was adopted by The International ARM Epidemiological Study [31] to reinvent AMD diagnosis. The grading procedure was described by nominal or modest non-exudative age-related macular changes. According to the ARM criterion, the presence of retinal pigment epithelium (RPE) atrophy was requisite to signify the manifestation of wet AMD. Another rigorous classification protocol has been described in the Age-Related Eye Disease Study (AREDS). This study divided the occurrence of AMD into four classes based on protein drusen attributes. Drusen were categorized based on its average diameter into small (63 μm and less), intermediate (63–124 μm), and large (125 μm and more). According to the AREDS, class 1, or no AMD, specifies that there exist less than five small drusen; class 2, or early AMD, specifies that there exists many small drusen or few intermediate-sized drusen; class 3, or intermediate AMD, is characterized by multiple intermediate drusen or few large-sized drusen and non-foveal GA without pigmentary atrophy; and class 4, or advanced AMD, is denoted by foveal GA and pigmentary atrophy, vision impairment due to CNV occurrence, and visual acuity lower than 20/32 in either eye [32,33]. A non-clinical system has been developed by Klein et.al. It bases its categorization on variations in demography and involves phenotypic and genetic considerations [34].

## 3. Advanced Therapeutics and Delivery Strategies for nAMD

**Figure 2:**
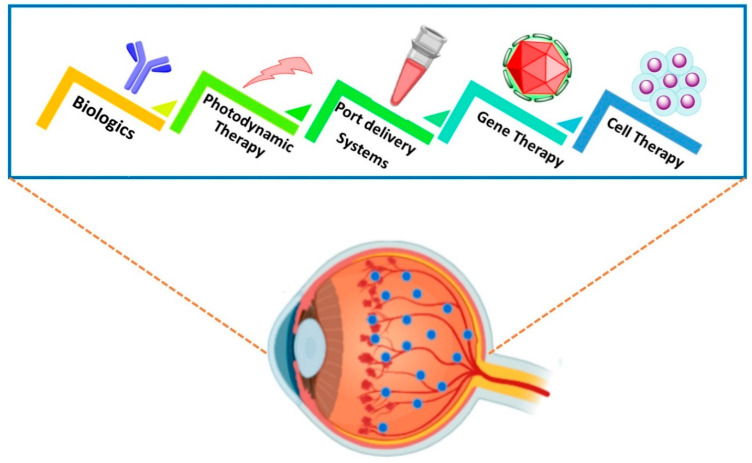
Ocular therapeutics and molecular delivery strategies for neovascular AMD.

In recent years, management of nAMD has undergone significant advances. In this section, recent progress on therapeutics and molecular delivery strategies for nAMD are highlighted. Various therapeutic and delivery strategies are shown in Figure 2.

### 3.1. Anti-VEGF Therapy and Its Delivery Strategies

Research has reported that VEGF is the most prominent angiogenic factor implicated in wAMD pathogenesis. VEGF is an essential cellular cytokine of the endothelium responsible for angiogenesis in the eye [35,36,37]. Studies reveal that VEGF protein concentration in AMD afflicted patients is significantly higher than in healthy individuals. This is indicative of the association between VEGF overexpression and AMD development [38]. Several anti-VEGF agents and their formulations have been developed and FDA approved, while others are in pre-clinical and clinical trials. The most investigated VEGF inhibitors that have revolutionized the therapy for neovascular AMD patients are Bevacizumab [39] and Ranibizumab [40]; other molecules, such as Aflibercept [41], Pegaptanib [42], and Brolucizumab [43] followed later. Table 1 summarizes the molecular targeted strategies for nAMD treatment.

#### 3.1.1. Bevacizumab

Bevacizumab (Avastin^®^) is a humanized full-length monoclonal immunoglobulin G (IgG1) antibody that is manufactured by recombinant DNA technology (~150 kDa) and is known to actively bind with high affinity and hinder the biological functioning of all VEGF isoforms. Bevacizumab received its first approval from the US FDA in 2004 as a therapeutic monoclonal antibody (mAb) to be administered in metastatic patients afflicted with colorectal cancer [40]. It was recognized as a potential AMD therapeutic by the US FDA in 2006 for off-label use [66]. There has been tremendous ongoing research for its efficacious administration through topical, intraocular, and intravitreal routes [67,68,69]. Sousa et al. manufactured poly (D, L-lactide-co-glycolide) (PLGA) nanoparticles encapsulating Bevacizumab to significantly augment its shelf-life without compromising the biological activity [70]. Furthermore, bevacizumab-loaded NPs were freeze-dried to improve their physicochemical stability. Fluorescence spectroscopy and in vitro bioactivity assays confirmed that the protein secondary structure initially underwent an alteration in conformation post encapsulation, but regained its confirmation upon release [70].

To enhance Bevacizumab VEGF inhibition and ocular bioavailability, Zahang et al. manufactured Bevacizumab loaded PLGA NPs that considerably increased the residence time of the formulation in the ocular fluids. Further research asserted that the NPs did not exhibit any form of cytotoxicity in murine retinal endothelial cells. In vitro analysis suggested that the formulated Bevacizumab was more efficacious in hindering VEGF-induced angiogenesis than Bevacizumab alone. The augmented anti-angiogenic effect for CNV treatment was confirmed through in vivo studies [71]. In another study, Bevacizumab was optimally loaded into nanoliposomes. It was observed that the optimization of encapsulation enhanced the protein’s stability. Though studies revealed that the release was slower than expected, the formulation exhibited a sustained release profile with minimal toxicity in retinal endothelial cells and no toxicity in human umbilical vein endothelial cells (HUVECs) [72].

Llabot et al. [73] developed Bevacizumab and suramin (anti-angiogenic agent) encapsulated in albumin NPs. The formulation was stabilized by Gantrez^®^ ES-425 and Glutaraldehyde. Further analysis revealed that the drug loading of NPs stabilized with Gantrez^®^ ES-425 was higher than those stabilized with Glutaraldehyde [39]. When Luis et al. manufactured Bevacizumab loaded human serum albumin (HSA) NPs for topical administration, the findings suggested that the formulated NPs exhibited improved efficacy in neovascularized mouse models compared to poly (ethylene) glycol (PEG) coated HSA NPs. The efficiency of antiangiogenic effect was estimated to be twofold in comparison to the unbound drug. Furthermore, PEGylated NPS did not significantly improve angiogenesis inhibition compared to the free recombinant mAb [67].

In another investigation, Ye et al. [74] developed Bevacizumab loaded PLGA microspheres to study the pharmacokinetic profile upon intravitreal administration in rabbits. The analysis revealed that the formulation exhibited a higher bioavailability of the active in the ocular fluids than the free drug. Furthermore, the results confirmed that the half-life of the microsphere was significantly higher in the vitreous humor (9.6 days) and aqueous humor (10.2 days) compared to Bevacizumab solution in the vitreous humor (3.91 days) and aqueous humor (4.1 days). Rauck et al. prepared Bevacizumab encapsulated PEG–poly–(serinol hexamethylene urethane) thermo-responsive hydrogel for intravitreal delivery. Analytical studies revealed that the hydrogel could be easily administered via a 31-gauge needle. In vivo studies suggested that the formulation exhibited minimal cytotoxicity and inflammation. The active had a sustained release profile for over 63 days. The protein concentration was maintained fivefold than the unbound drug solution [75].

#### 3.1.2. Ranibizumab

Ranibizumab (Lucentis^®^) is a fragment of humanized monoclonal immunoglobulin G (IgG1_k_) antibody [44] which can effectively bind to all VEGF-A isoforms, including VEGF_165_, VEGF_121_, and VEGF_110_ [45]. The mAb genetic composition includes a non-binding human sequence and a high-affinity murine epitope. Such a construction reduces the antigenicity of the antibody in humans [76]. Replacement of specific amino acid sequences augmented the binding efficacy of Ranibizumab to VEGF surface receptors hundredfold compared to Bevacizumab [77]. As it is an antibody fragment, Ranibizumab has a lower systemic half-life due to its enzymatic degradation. This consequence is consistent as full-length antibodies have a higher residence time than antibody fragments [40]. Ranibizumab received its first approval from the US FDA in 2006 as a therapeutic mAb for nAMD patients. A 12-year clinical cohort study on the patients with Ranibizumab and Aflibercept concluded that the visual outcomes positive within the first 2.0 years after treatment. However, within 8.7 years the visual outcome downgraded to poor vision [78].

Yan et al. fabricated Fe_3_O_4_/PEGylated PLGA NPs hybridized with Ranibizumab and HUVEC cells identified to determine the long-term anti-angiogenic activity using tube formation in Matrigel assay. It was observed that such hybridization yielded improved inhibition of angiogenesis and no cytotoxicity compared to unloaded Fe_3_O_4_ NPs [79]. There were attempts to improve the drug release from solid lipid implants (SLIs); in one study the authors reported that Ranibizumab was released for several months and no changes were found in the secondary structure [80]. In another investigation, Tanetsugu et al. formulated a sustained release PLGA microparticle system encapsulating Ranibizumab-biosimilar. Through assay of tube formation by HUVEC cells, further pharmacokinetics revealed that the biosimilar had a prolonged release of ~80% over three weeks upon intraocular administration [81]. In a comparative study, Antoszyk et al. [82] compared the therapeutic activity and side-effects of the combination of Ranibizumab with Verteporfin and PDT alone. In a randomized two-year controlled evaluation, it was observed that 88% of both Ranibizumab and PDT treated patients with CNV experienced improved visual acuity at the end of 24 months. Studies reported that the accumulation of retinal fluid and lesion proliferation in patients with combination therapy was significantly diminished. Furthermore, the occurrence of severe adverse effects was lower in the Ranibizumab and PDT treated patient cohort than in the PDT cohort.

#### 3.1.3. Aflibercept

Aflibercept (Eylea^®^) is a fusion protein composed of binding domains of VEGF receptors 1 (VEGFR-1) and 2 (VEGFR-2) fused with the Fc region of human immunoglobulin G (IgG1) antibody [83]. It can bind with high-affinity to the VEGF-A, B, C, and D isoforms [46] and Placental growth factor (PGF) [84]. The significant advantages of Aflibercept include its high VEGF binding affinity and a longer systemic half-life upon intravitreal administration [85]. A similar novel anti-VEGF agent, conbercept was developed. Its protein structure consisted of an antibody fragment derived from the Fc region of immunoglobulin 1 (IgG1) and extracellular domain 2 of VEGFR-1 and extracellular domains 3 and 4 of VEGFR-2. Studies suggest that conbercept can potentially inhibit VEGF-A, B, and C along with an improved VEGF binding affinity [86]. Aflibercept received its first approval from the US FDA in 2011 as a therapeutic molecule to be administered in nAMD patients [87].

Liu et al. developed a drug formulation that could exhibit controlled and prolonged release for over 180 days. Therefore, Aflibercept-incorporated PLGA microspheres were encapsulated within a poly (ethylene glycol)-*co*-(l-lactic acid) diacrylate/*N*-isopropylacrylamide thermoresponsive hydrogel. Studies suggested minimal cytotoxicity from the degraded by-products. Furthermore, enzyme-linked immunosorbent assay (ELISA) revealed that the bioactivity of Aflibercept was maintained at a considerable therapeutic level at all times [88].

#### 3.1.4. Pegaptanib

Pegaptanib (PGB) (Macugen^®^) is the PEGylated form of a neutralizing RNA aptamer. Aptamers are oligonucleotide ligands that can selectively bind to specific intra- and extra-molecular targets with very high-affinity [47]. The design of PGB specifically targets the VEGF_165_ isoform to inhibit cell proliferation and thereby block choroidal neovascularization [46]. It received its first approval from the US FDA in 2004, however it is no longer a clinical recommendation as more efficacious molecules entered the market subsequently [89]. Pharmacokinetic studies display that PGB has a mean plasma half-life of ~8 days for an intravitreal dose of 0.3 mg. Furthermore, no significant systemic accumulation was observed post administration of 8 doses [90]. In underweight patients with low creatinine levels, the PGB plasma concentration was found to be the highest [91]. Cook et al. [92] formulated a PLGA based microsphere encapsulating PGB by an emulsion solvent evaporation methodology. The microspheres were assessed for their drug loading, in vivo activity, and in vitro release profile. Studies suggested that the entrapment efficiency was significantly high (~97%) and the drug is not degraded during incorporation and subsequent release. The in vivo analysis revealed that the plasma concentration was appreciably maintained at required levels over multiple weeks.

#### 3.1.5. Brolucizumab

Brolucizumab (Beovu^®^) (ARTH 258) is a single-chain antibody fragment (scFv) [48] with the smallest molecular weight (~26 kDa) discovered as an anti-VEGF agent [93]. It binds to all VEGF isoforms with high affinity and subsequently inhibits the cellular proliferation [43]. A relatively new biologic, it received the US FDA approval in 2019 as a therapeutic molecule for the treatment of exudative AMD, diabetic macular edema (DME), and retinal vein occlusions. This approval was primarily based on preliminary results furnished in both of the phase III clinical trials [94]. The primary advantage of Brolucizumab is its small size compared to other anti-VEGF drugs, for example, 115 kDa (Aflibercept) and 48 kDa (Ranibizumab). This property enables its solution concentration up to 120 mg/mL and permits administration of 0.05 mL doses for 6 mg of drug delivery. In a comparative analysis, this dose is equivalent to ten-fold the dose of 2 mg Aflibercept and twenty-fold the dose of 0.5 mg Ranibizumab [95]. From a theoretical perspective, high VEGF inhibitor concentrations in ocular fluids involve higher clearance time and consequently prolonged duration of action. From the clinical evidence perspective, the 96 weeks of phase III HAWK and HARRIER trials revealed that Brolucizumab was well tolerated and found to be effective in nAMD [80]. In recent case studies, several side effects have been reported, such as intraocular inflammation (IOI), retinal vascular occlusion (RO), and retinal vasculitis (RV) [96]; in the same study, patients reported a decline in visual acuity (VA). Continuous updates on side effects are found on a dedicated information resource website [97].

#### 3.1.6. Bispecific Antibodies

Bispecific antibodies are of special interest in ophthalmology due to their dual antigen domain binding capabilities [98,99]. Table 2 lists the bispecific antibodies for AMD therapy. IBI302 is a novel recombinant human anti-VEGF and anti-complement bispecific fusion protein for AMD that collectively inhibits proliferation of VEGF-mediated signaling pathway and reduces the inflammatory response mediated by complement activation. The pharmacokinetic (PK) properties in rhesus monkeys showed (0.5 mg/eye intravitreal) activity over 504 h [55]. In the phase-1 study, IBI302 IVT injection displayed improved vision and reduced vision with good safety and tolerability in 31 patients after 6 weeks of administration. In another study, Faricimab (RO6867461; RG7716), a bispecific domain-exchanged (crossed) monoclonal antibody (CrossMAb) was studied in the CNV-induced model after intravitreal delivery. The mechanism of action includes binding and neutralizing both VEGF-A and ANG-2 [56]. It is interesting to note that angiopoietin-2 (ANG-2) specific molecules gained momentum in recent times in clinical trials [100].

#### 3.1.7. Abicipar Pegol

Abicipar pegol (MP0112) is a newer drug that belongs to the class of designed ankyrin repeat protein (DARPin). It shares its structural identity with ankyrin, which can bind to all VEGF-A isoforms and is a potential candidate in AMD therapy [49]. Recent results from phase II clinical trials suggest that the efficacy of abicipar is similar to Ranibizumab in improving visual acuity over 20 weeks. Furthermore, analytical studies indicate that an 8–12-week regime of abicipar may provide the same therapeutic benefit as a 4-week regime with Ranibizumab. Currently, phase III clinical trials of abicipar are underway to confirm the above claim. Though results show promise, the post-administration care required to treat and prevent ocular inflammatory responses makes it ambiguous [101].

#### 3.1.8. Limitations of Anti-VEGF Therapy

Though intravitreal administration of VEGF inhibitors is the first line of drugs utilized in AMD therapy, there are several limitations. Furthermore, this therapeutic modality necessitates frequent monthly or bi-monthly delivery [23] as the administered drug has a short retention time, primarily due to the high protein clearance rates from the ocular humor [85]. Intravitreal injections of VEGF inhibitors have been reported to present decreased bioavailability of the drug due to restricted ocular space, lachrymal drainage, and ocular enzymatic degradation [102,103]. Studies suggest that several ocular complications such as intraocular hemorrhage, RPE tear, increased intraocular pressure, inflammation, vitreous bleeding, and retinal detachment may occur [104]. KG Falavarjani et al. summarized the frequency of these adverse events, i.e., Endophthalmitis (0.019 to 1.6%), intraocular inflammation (bevacizumab injection 0.09–0.4%), Rhegmatogenous retinal detachment (0 to 0.67%), and ocular hemorrhage (10% of injections) [105]. As sterility is a pre-requisite to delivering intravitreal and intraocular injections, its absence can lead to pathogenic infections and endophthalmitis. In severe cases, a vitrectomy may become a necessity [22]. Several systemic complications have also been associated with VEGF inhibitors, including myocardial infarction, stroke, and hypertension [106], however these events are extremely rare. Marc Schargus and Andreas Frings summarized problems associated with intravitreal administration [107]. This is attributed to the mechanical trauma of the meshwork, interfering with fluid outflow, and the accumulation of protein aggregates from the injections [108,109,110,111,112,113]. Several studies found endophthalmitis and a series of inflammatory reactions. However, some of these challenges were addressed by prefilled syringes [114,115,116,117,118]. Silicon oil droplets were even found 12–18 months after intravitreal injections; in about 5% of cases in the USA they were causing problems [119]. In a comparative study, small volume syringes were evaluated for their accuracy and precision, only one commercial syringe was found to be sufficiently accurate to deliver the right amount [120]. Furthermore, a clinical setting study revealed dosage variations in 669 injections. The target volume of 50 µL deviated by up to 25%. The pharmacodynamic variations could be attributed to the dosing variations [120]. Research [121] suggests that long-term injections or injections that require repeated administration can significantly burden the patient and cause distress from a financial perspective. Lucentis^®^ (Ranibizumab) costs about USD 2000 [122].

#### 3.1.9. Bioavailability, Dosing, and Pharmacokinetic Considerations of Anti-VEGF Agents

Subrizi et al. proposed a simple equation based on steady-state concentration at the target site, drug clearance, dosing interval, and bioavailability parameters [123] to estimate the dose required for rabbits in the formulations for intravitreal drug doses for different time intervals. Moreover, the equation was successfully applied to published in vivo data for various drug delivery systems [123]. It is evident that highly potent molecules are required for long-acting formulations, ant that exploratory studies often ignore this scientific observation. Interestingly, another pharmacokinetic (PK) simulation based on diffusion model estimated the concentrations of Bevacizumab, Ranibizumab, Rituximab, Conbercept, and Aflibercept and the observations well correlated with the available pharmacokinetic data. Furthermore, their model predicts that a few milligrams for 12 months is enough to maintain therapeutics vitreal concentrations in the range of 0.1 to 0.2 µM [124]. Table 3 summarizes the relationship between drug dose and dosing frequency for drugs with different target concentrations from pharmacokinetic simulations.

### 3.2. Novel Therapeutic Agents for AMD: Beyond Anti-VEGF Therapy

#### 3.2.1. Platelet-Derived Growth Factor (PDGF) Inhibitors

Platelet-derived growth factor is an influential cellular mitogen that plays a significant role in retinal pericytes survival and functioning [125]. The development of pegpleranib and rinucumab was targeted with the intention of combined inhibition of VEGF and PDGF receptors [126]. However, phase III results state that the combined therapy does not yield any additional benefits than VEGF inhibitors [127]. This failure can be partly ascribed to the complex and multifactorial action of PDGF on ocular angiogenesis. Therefore, by inhibiting PDGF, both the pericytes functioning and cellular proliferation are blocked. However, inhibiting the pericytes can significantly diminish the stabilization function and lead to dilation of capillaries with increased exudate formation and severe neovascularization [128].

#### 3.2.2. Pigment Epithelium-Derived Factor (PEDF) as a Treatment Agent

Pigment epithelium-derived factor is a low molecular weight (50 kDa) glycoprotein [129] that is secreted by the RPE cells to regulate the activity of selective metalloproteinases [130] and block the angiogenic stimuli induced by hypoxia-induced factor 1 (HIF-1) and VEGF levels [131]. Though it shares its structural identity with serine protease inhibitors, it does not block proteases. Studies suggest that PEDF is critically involved in neuroprotective functions and neuronal differentiation [129,132,133]. Further research revealed that PEDF is a potent inhibitor of cell proliferation, permeation, and migration [134].

As CNV is the lead cause of AMD manifestation, the role of PEDF has been thoroughly investigated. A spectrum of studies reveals that the diminishing levels of PEDF in the RPE and choroidal cells is the primary factor responsible for causing an imbalance between the angiogenic and anti-angiogenic processes. This disturbed equilibrium thereby leads to CNV development [135].

#### 3.2.3. Angiopoietin Inhibitors

The function of angiopoietins (ANG) on CNV is complex; however, studies reflect that it majorly depends on the composition of inflammatory factors in the ocular environment [136]. Theoretically, ANG-1 is present in copious quantities in vascular surroundings, and its primary function is the maintenance of homeostasis by inhibition of cellular proliferation, permeation, and exudation. Researchers have also found that it has the capacity to diminish fibrosis [137]. On the contrary, the bioactivity of ANG-2 is much more complex to understand. ANG-2 plays a significant role in vascular stabilization. It has been detected that, in ocular surroundings with high ANG-2/VEGF ratios, cell proliferation is hindered [138]. However, when the inflammatory cytokines have exerted their action, ANG-2 behaves as an antagonist and functions with the VEGF in synergy to induce CNV [139].

Faricimab is a novel bispecific mAb [140] that can target both VEGF-A and ANG-2. From the results of the 12–16-week regime tested in phase II trials (STAIRWAY), it was concluded that the drug loading after every four weeks was similar to that of Ranibizumab. Currently, there are two phase III trials underway (TENAYA-NTC03823287 and LUCERNE-NTC03823300) [48]. Nesvacumab is another monospecific mAb that binds to ANG-2. It was evaluated as a supplementary therapy to Ranibizumab; however, the phase II trials were rejected as the molecule failed in efficacy testing [43].

#### 3.2.4. Anti-Inflammatory Agents and Other Small Molecules

Another critical factor that has been evidenced in the pathogenesis of AMD is the intraocular inflammatory signals. This indicates that certain anti-inflammatory agents may play a role in the regression of the macular degeneration. The results from Blue Mountains Eye study [141] concluded that there was no apparent association between AMD prevalence and the administration of systemic non-steroidal anti-inflammatory drugs (NSAIDs).

Triamcinolone acetonide is a widely studied, anti-inflammatory agent; its mechanism is not fully elucidated. It is extensively used in diseases such as Scleritis, Uveitis, Diabetic Macular Edema, and Exudative Macular Degeneration in combination with other agents or therapies [142,143]. Studies suggest that triamcinolone acetonide (TCA), an anti-inflammatory and angiostatic agent may effectively block CNV [144,145]. A placebo-controlled clinical trial conducted in Australia concluded that no specific relief from loss of visual acuity in patients with classical CNV was derived with a single triamcinolone dose. However, the size of the vascular membrane was smaller than the control cohort [146]. Current research investigates the prolonged anti-angiogenic activity of intravitreal triamcinolone with Photodynamic therapy (PDT), explained in detail in Section 3.4 [57]. However, specific incidences have severe adverse effects, including increased ocular pressure, cataract progression, pseudo-endophthalmitis, and retinal tear have been reported [147].

Dexamethasone is a highly potent anti-inflammatory agent widely used in ocular applications. In a clinical non-comparative case study, 27 patients were given intravitreally using a 30-gauge needle. Dexamethasone Phosphate (0.2 mg) and Bevacizumab (1.25 mg) during a 6-month period, the non-comparative study concluded that the combination provided benefit in macular edema and improved visual acuity [148]. In another clinical study, dexamethasone intravitreal implant (DXI; 700 μg, Ozurdex; Allergan, Irvine, California, USA) after monthly Ranibizumab injections reduced sub-retinal fluid (SRF) and central retinal thickness (CRT) [149]. The Young Ophthalmologists Reviews Study Group (YORSG) comprehensively reviewed the clinical benefits of intravitreal implant and therapeutic potential of dexamethasone, clearly indicating the therapeutic benefits [150]. However, another clinical study reported no additional benefits in combination with Ranibizumab or Ranibizumab monotherapy, hence a lack of clear direction, requiring further studies to establish the synergistic or additive benefits [151].

Another angiostatic steroid and glucocorticoid analog [152], anecortave acetate (Retaane, Fast track designation FDA), has been investigated to treat neovascular AMD. It is known to block choroidal angiogenesis by inhibiting proteases responsible for cellular migrations [153]. An interesting observation concludes that it does not hinder retinal angiogenesis during its inhibitory activity [152]. Compared to triamcinolone, adverse side effects such as cataract development and increased ocular pressure are not observed. Furthermore, anecortave non-specifically blocks angiogenic functions that are induced by various endogenous stimuli. Current clinical trials are directed towards the assessment of its efficacy in combination with Visudyne^®^ (PDT) [58].

Sorafenib is an oral TKI (tyrosine kinase inhibitor) that suppress VEGF signaling by inhibiting VEGF receptors 1 and 2 (VEGFR). In an off-label exploratory study, two patients were given oral sorafenib (200 mg) along with the intraocular injection of Ranibizumab, and the study revealed a marked improvement in visual acuity in both patients. However, no other studies followed this observation further [59]. Sunitinib malate (Sutent^®^) is also a TKI molecule that acts on several TK receptors responsible for neovascularization [154]. It is a malate salt of that parent molecule with higher solubility and bioavailability. Graybug Vision, Inc., (Redwood City, California, United States) a clinical-stage biopharmaceutical company, studied the therapeutic potential attentively [155]. Two sustained-release depots formulations are under development [60,61].

A recent proof of concept clinical research study (NCT03022318, NCT03023059) concluded that levodopa oral formulation was safe and well-tolerated. In the case of patients taking anti-VEGF injections, the injection frequency was reduced. The application of levodopa as an adjuvant needs further evaluation due to the low sample size and limited racial diversity [156]. Levodopa acts as a ligand for G-protein coupled receptor (GPR143), upregulates the PEDF, and downregulates the production of VEGF [63,64,65].

### 3.3. Port Delivery System (PDS)

**Figure 3:**
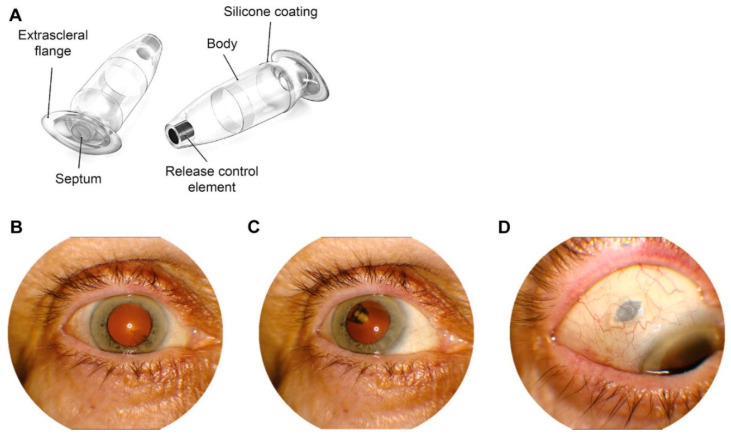
Port Delivery System with ranibizumab (PDS) implant. Port Delivery System with ranibizumab implant (**A**); Patient images from a PDS-implanted patient with: (**B**) eye in primary position (implant not visible); (**C**) eye looking up with implant visible through dilated pupil; and (**D**) eye looking down to visualize PDS septum. Reprinted from ref [158] with permission from Elsevier.

One nAMD therapeutic system that has garnered much attention in the past few years, due to its promising ability to reduce the frequency of anti-VEGF drug administration, is an implantable Port Delivery System (PDS). Figure 3 shows a Port Delivery System with Ranibizumab implant in the eye after implantation. It is designed to allow the sustained and controlled release of agents that effectively neutralize VEGF [95]. The most investigated implant, Ranibizumab PDS (Ranibizumab-PDS) is in late phase clinical trials. It is a small, refillable ocular implant with high durability that can effectively dispense Ranibizumab when the reservoir is positioned in the vitreous cavity [157].

The PDS is implanted in the incised sclera and pars plana. The system is composed of a self-sealing septum (drug reservoir for refilling) in the center of an extra scleral flange to fix the PDS in the sclera, and a porous metal release element that enables passive diffusion of the drug. This owes to the development of a concentration gradient in the vitreal cavity. The implant aims to use a designed Ranibizumab formulation which is different from Lucentis^®^ [158]. The most recent evidence of Ranibizumab-PDS clinical trials reflects its promise to improve visual acuity in nAMD patients [159]. Given the sustained delivery of Ranibizumab from the implant in MARINA and ANCHOR clinical trials, Ranibizumab is a potential anti-VEGF agent for PDS therapy [160,161].

The results of phase II clinical trials (Ladder) reflect that PDS with 100 mg/mL the formulation is not inferior to monthly Ranibizumab intravitreal injections in reference to improvement in visual acuity [158]. These promising results led to phase III clinical trials. To date, PDS has undergone several modifications [162], this includes the improved surgical protocol during implantation with laser-based ablation of the pars plana at the incision region in the sclera that reduces the incidence of vitreal hemorrhage [163]. This indicates that PDS can indeed transform the therapeutic modality for nAMD. The ongoing phase III clinical trials can provide us important insights into the effectiveness of PDS and how it can potentially replace the need for frequent anti-VEGF administration. A.M. Khanani et al. summarized and provided the PDS technology evaluation report [164].

### 3.4. Photodynamic Therapy and Its Delivery Strategies

Photodynamic therapy (PDT) involves activation of a photosensitizer by photon irradiation that generates reactive oxygen species (ROS) that selectively occlude CNV in the target ocular segment [106]. It has found extensive applications in exudative AMD treatment before the rise of anti-VEGF therapy. Visudyne^®^ is a liposomal formulation of a hydrophobic photosensitizer, Verteporfin that was approved for nAMD administration by the US FDA in 2000 [165]. Photodynamic therapy is generally used as alternative therapy or adjunct therapy along with anti-VEGF injections according to various country guidelines [166,167]. Table 4 summarizes the photodynamic therapy and delivery strategies for nAMD.

Upon intravenous administration, the PS (verteporfin ~689 nm) accumulates in the irregular blood vessels. When activated with infrared radiations, the PS undergoes a chemical alteration that generates cytotoxic ROS radicals that block choroidal neovascularization. This results in cessation of bleeding and selective occlusion of the newly formed blood vessels along with decreased exudate formation and significant damage to RPE may occur [187]. Another formulation developed by Nikolai et al. studies the effect of verteporfin incorporated in cationic liposomes (CL-VTP) in augmenting CNV occlusion. In a comparative analysis, Visudyne^®^ led to a size reduction in CNV (~56%) compared to CL-VTP (~53%). Furthermore, cationic liposomes exhibited lower retinal deterioration compared to Visudyne^®^. When extended, studies suggest that paclitaxel or succinyl-paclitaxel-loaded CL can significantly inhibit angiogenesis in rat models [168].

Li et al. have formulated liposomal Hypocrellin B (HB) for the therapy of exudative AMD [169]. The photosensitizer is extracted from the fungal sacs of *Hypocrella bambuase.* It is known to absorb radiations between 450–600 nm and potentially lead to the generation of reactive radicals that penetrate the tissue (~1 mm). When the formulation (1 mg/Kg) is intravenously administered and irradiated with yellow light in rat models, the CNV area is significantly reduced with very low tissue damage. The further evaluation suggests that HB (half-life of 2.31 ± 0.46 h) is cleared from systemic circulation and exhibits minimal skin phototoxicity after 24 h [169].

In another investigational study, Shimazaki et al. has evaluated the pharmacological effect of sub-micron sized liposomes encapsulating edaravone (3-methyl-1-phenyl-2-pyrazolin-5-1) to treat induced retinal impairment in mice [170]. Prior to the experimentation, the retinal damage induced by light exposure was assessed by documenting the scotopic electroretinogram. The results of the exploratory study suggested that when the formulation was administered as an eye drop, the topical dose caused a reduction in the thickness of the outer nuclear layer. Furthermore, when compared to the free drug, it was observed that the liposomal drug exhibited improved inhibition of light-induced ROS generation and consequent cellular death. Studies also suggested that the formulation showed little to no cytotoxicity, thereby a potential therapeutic strategy for nAMD treatment [170].

In another exploratory study, Lajunen et al. has investigated the therapeutic efficacy light-activated nanoparticles can render when administered in the eye [188]. In one study, liposome-based drug carriers have been formulated that incorporate gold nanoparticles (AuNPs) for external stimuli, light-triggered drug release. The study revealed that controlled cytosolic drug release was observed in presence of light. In the absence of light or AuNPs, no release was reported. In addition, no specific cases of cellular toxicity were observed, making it a potential candidate for AMD therapy. According to the publication, it is suggested that the AuNPs absorb the near-infrared radiation and the released energy, in the form of heat, reduces the impermeable character of the liposomal bilayer leading to drug release [171].

Following this analysis, the AuNPs were substituted indocyanine green (ICG). It was noted that the ICG plays an important role in light activate release as it improves the release of the drug, enhances the stability, and increases the possibility of controlling the liposomal size along with speedy regulatory approval owing to the clinically approved imaging agent [189]. Lajunen et al. have also studied the interactions between the lipid bilayer, the ICG, and the PEG coating on the liposomal stability. According to the preliminary data, it has been suggested that the localization of the ICG considerably alters the liposomal structure and therefore may affect the release of the drug. Currently, in-depth studies to identify and determine the exact mode of action and effect of the ICG on the drug release is in progress [172].

Jia-Lin et.al. has investigated combination therapy of targeted PDT and anti-VEGF (sorafenib) on laser induced CNV rat model. In this study, photocyanine (photodynamic activity) and sorafenib (novel multi-kinase inhibitor, anti-VEGF activity) encapsulated (90% encapsulation) into RGD-modified liposomes and further targeted to integrin (arginine-glycine-aspartic acid (RGD) receptor) overexpressed CNV endothelial and RPE cells. In vivo studies showed reduction in CNV area and leakage of fluorescein in laser-induced CNV rat models with high safety profile [173].

As PDT exhibits CNV specific targeting capacity, theoretically, it should treat the retina with minimal damage. However, studies suggest that even though PDT reduces drug administration frequency, loss of vision and adverse side effects, including RPE and photoreceptor damage [22], have been reported. Furthermore, research reveals that though PDT leads to lower retinal deterioration compared to laser therapy, its efficacy is significantly limited [190].

These complications have led to studies that evaluate the combined efficacy of anti-VEGF drugs and PDT as primary and adjuvant therapy, respectively [191]. However, the results of these comparative studies lead to ambiguity. Some research groups have concluded that intravitreal therapy of VEGF inhibitors (Bevacizumab or Ranibizumab) is potentially more efficacious compared to PDT alone or a combination of PDT and anti–VEGF agents [192,193]. On the contrary, Gao et al. has concluded that the combination of Visudyne^®^ based PDT and VEGF inhibitors is more effective than anti-VEGF monotherapy. Furthermore, the combination can potentially reduce the frequency of intravitreal administrations [174]. With such results, more targeted research and clinical efficacy studies are required to conclude the efficiency of therapeutic modalities for the treatment of nAMD.

### 3.5. Radiation Therapy

Though administration of anti-VEGF agents remains the primary standard of care for neovascular AMD, its need for frequent administration and ineffectiveness in some patient groups necessitate the introduction of a novel therapeutic regime [22]. Radiation therapy can cause irreparable damage to the protein synthesis function, restricting further cell proliferation while still retaining cellular integrity [194]. Although clinical results suggest that radiotherapy can potentially treat neovascularization, its chances to compete with VEGF inhibitors are low [195,196]. To overcome the limitations posed by EBT, epimacular brachytherapy was developed to deliver radiation via the intraocular route. Brachytherapy refers to the delivery of the radiation directly to the affected tissues in a way that places the source of light in very close proximity to the CNV region [194]. The strontium-90/yttrium-90 source generates β-radiation for therapy [197]. JR Evans et al., in their invention review, summarized the 18 relevant studies (2340 people) with wet AMD. Studies investigated external beam radiotherapy (15) and internal radiotherapy (3) (brachytherapy). In a 12 month follow up study, radiotherapy was compared with no radiotherapy or a sham treatment, and no consistent evidence on therapeutic benefit was revealed [198]. 

Studies suggest that, though VEGF inhibitors exhibit rapid therapeutic activity, it is restricted by several limitations. However, this drawback can be potentially overcome by the concurrent administration of anti-VEGF drugs and radiotherapy. As radiotherapy involves a major time lag and a prolonged duration of action, it can potentially augment the therapy of AMD in theory [199]. However, clinical trials and comparative studies negate such predictions and provide ambiguous conclusions. Recent clinical trials suggest that monotherapy of VEGF inhibitors is still superior to the combination therapy of anti-VEGF drugs and radiation therapy. Such results necessitate deeper understanding and large-scale trials to obtain accurate therapeutic results [200]. Table 4 summarizes the combination therapy of anti-VEGF agents with radiation for nAMD. In a 12 month follow-up study, comparing radiotherapy combined with anti-VEGF and anti-VEGF alone concluded that effectiveness for treating wet AMD was uncertain as to whether radiotherapy on its own, or with eye injections of anti-VEGF, was more beneficial [198].

### 3.6. Gene Therapy and Its Delivery Strategies

Recent years have seen a bloom in the research of gene therapy in hereditary disorders. These predominantly include Leber’s congenital amaurosis (LCA), choroideremia, retinitis pigmentosa (RP), and Stargardt’s macular dystrophy [201]. The success of gene therapy in LCA has resulted in the experimentation of gene therapy for AMD patients. Studies show that a single time genetic therapy in AMD afflicted individuals to transduce RPE cells holds promise to induce the production of anti-angiogenic agents in vivo [202]. Table 4 summarizes the gene therapy and delivery strategies for nAMD.

#### 3.6.1. Description of Viral Vectors

Several viral and non-viral gene administration techniques have been developed over the past few years. The viral vectors commonly employed adeno associated virus (AAV), adenovirus, and lentivirus [180]. The systematic selection of viral vectors plays a significant role in the intended application, and this is based on the concurrent effect of tissue physiology, the capacity of the vector to engage in cloning, and safety concerns related to inflammation. Studies suggest that while the first-generation adenoviral vectors have restricted applications due to induction of inflammatory responses, the second-generation vectors have low immunogenicity. The third-generation adenoviral vectors are by far the safest, with a high cloning capacity and the ability to effectively transduce a wide variety of cell classes [203,204].

Various investigations and case studies have reported that AAV vectors are less immunogenic, thereby increasing their promise as a viral carrier for AMD therapy. Unlike the adenoviral vectors, AAV vectors provide availability of several serotypes with each exhibiting tropism [205]. For instance, studies have proven the use of AAV2 for transduction in skeletal muscles and retinal membranes [206]. Clearly, AAV vectors have attracted immense attention for retinal therapy. This is mainly due to the vector’s non-integrating features, the low potential of inducing inflammation, low cytotoxicity specifically in the retinal periphery, non-pathogenicity, its capability to transduce non-reproducing cells and its excellent demonstration of safety in human clinical trials [207,208]. Table 5 shows completed and ongoing gene therapy clinical trials for nAMD. Along with its various advantages, it does have some shortcomings. It presents a restricted transgenic capacity (~4.5 kb) and exhibits the risk of rapid elimination due to the humoral immunity in individuals who have been previously exposed to this viral vector [209]. Even with its drawbacks, the immunogenic risks of AAV vectors are much lower when administered in immune-privileged retinal tissues [210].

Lentivirus vectors have undergone tremendous research in the past few years for their utility in genetic therapy, in particular, those derived from the human immunodeficiency virus 1, simian immunodeficiency virus, feline immunodeficiency virus, and equine infectious anemia virus [211]. They have a lower cloning capacity (~10 kb) compared to adenoviral vectors (~35 kb) [212]. These vectors have found extensive applications in therapeutic regimes where vector genome integration in the parent genome is requisite [213]. Recently, it has been noted that these vectors can effectively transduce quiescent cells, apart from reproducing tissues, while improving the safety profile [214]. Meanwhile, studies report that the newly developed non-integrating lentivirus vectors can make considerable progress towards diminishing genotoxic adverse reactions [215].

#### 3.6.2. Mode of Administration

Though studies reveal the possible immunogenicity due to viral vectors, the eye, being relatively immune-privileged, blocks any elicitation of immune responses and restricts the systemic diffusion of introduced genetic material. Furthermore, the direct availability of introduced genes to target retinal cells and the capacity for non-invasive monitoring of therapy progression enables a more controlled therapeutic system [216]. Currently, there are two methods for viral vector delivery; they include intravitreal injection or pars plana vitrectomy (PPV) before administration of a sub-retinal injection [180].

Among the two, PPV prior to a sub-retinal injection of the vector encoding for the specific genes is preferred. Though PPV causes a temporary detachment of the retinal membrane, the invasive technique enables delivery directly to the target cells, improving specificity and selectivity. The viral particles ‘infiltrate’ the RPE cells, causing the host cells to transcribe and translate the administered genes into the desired therapeutic protein [217]. The other method involves the administration of an intravitreal injection into the vitreous humor. Although, this procedure is less invasive and presents lower procedure-related complications, the accessibility of the viral vectors to the target cells is potentially low [218].

#### 3.6.3. Viral Vectors for Gene Therapy

Several experiments have highlighted that certain endogenous factors are implicated in the pathogenesis of neovascular AMD. Due to their abnormal physiologic concentrations, the individual develops nAMD over time. This is primarily ascribed to the over-expression of VEGF, the deficient PEDF concentrations and the under-expressed extracellular domain of VEGF1 receptor. Other factors include endostatin and angiostatin [175].

##### Pigment Epithelium-Derived Factor Gene Therapy

The first genetic intervention developed for nAMD involved an intraocular delivery of the PEDF gene in patients progressing through advanced AMD. The phase I clinical trials of AAV-PEDF were published in 2006 [175]. As PEDF is a physiologic peptide integral in angiogenesis inhibition, it can play a crucial role in the nAMD therapy [219,220]. Campochiaro et al. assessed the efficacy and safety profile of E1-, partial E3- and E4-eliminated second-generation adenovirus vector that could induce expression of human PEDF. The analysis was conducted in 28 patients who were affected with advanced nAMD. Apart from the temporary ocular inflammation in seven patients, no deleterious side effect was noted. No concrete evidence of improved visual acuity was recorded upon sample administration. This was mainly attributed to the absence of control groups and the small patient groups used for assessment. However, it was noted that patients who received less than 108 particles have a decreased improvement in visual acuity compared to patients who received more than 108 particles. This result could possibly indicate the relationship between response and dose escalation [176]. At present, no concrete results are available to support this hypothesis.

##### VEGF Gene Therapy

Recent research is focused on the manipulation of soluble fms-like tyrosine kinase-1 (sFLT-1) for anti-angiogenic effect. It is an endogenous anti-VEGF agent that can effectively bind to VEGF-A receptors and block the interaction of VEGF protein and its endothelial receptor. Preclinical murine models have demonstrated inhibition in CNV development upon administration of recombinant AAV (rAAV) with sFLT-1 via a sub-retinal injection. This is attributed to the direct entry of viral vectors into the RPE cells that allow for transduction and subsequent expression of sFLT-1 via physiological protein synthesis mechanisms of the individual [177,178,179].

Avalanche Biotechnologies in association with Australian Lions Eye Institute has assessed the safety of sub-retinal injection of rAAV sFLT-1 [202,221]. In the phase I clinical trials, the 36 month follow-up concluded that no RPE cellular proliferation, retinal scar formation, or choroidal atrophy was noted. However, specific patient groups experienced conjunctival and retinal hemorrhage along with the progression of cataracts. These adversities were attributed to the complications associated with subretinal delivery. Furthermore, patients who received high doses of the sample required reduced doses of anti-VEGFs compared to the control groups. The promising results of the phase I initiated phase II clinical trials [202].

Similar to the results of phase I clinical trials, the phase II studies concluded that no specific systemic adverse reaction or ocular toxicity was observed [180]. Although there were two patients who reported the incidence of ocular inflammation and cataract formation [222], it was concluded that the side effect was related to the mode of viral vector delivery. The second-generation genetic therapy involves ADVM-022 and ADVM-032. Both employ vectors that can transfer anti-VEGF cDNA to the target cell for expression of Aflibercept and Ranibizumab upon intravitreal administration. ADVM-022 is currently under active phase I clinical trial (trail name: OPTIC, NCT03748784), an observational long term trail (OPTIC-EXT, NCT04645212) for wet AMD, the results are expected in early 2022. However, same molecule under phase II trial for Diabetic Macular Edema (DME) (trail name: INFINITY, NCT04418427) has recently been suspended after severe adverse effects were reported, such as decreased ocular pressure, inflammation, and loss of vision in the treated eyes of subjects [223,224,225].

Sanofi Genzyme has evaluated the intravitreal administration of AAV2-sFLT1 and showed a significant increase in gene expression at the human fovea. The sFLT1 is a fusion protein composed of the sFLT-1 domain2 and the Fc domain of IgG1. The chicken-β-actin (CBA) promoter, a fusion of the chicken-actin promoter and cytomegalovirus (CMV) immediate-early enhancer, was employed to cause higher expressions of the protein in the macaque Müller and ganglion cells after IVT injection [226]. Regenexbio is investigating the application of a novel AAV8 vector, RGX-314. It is designed to express a soluble fragment of anti-VEGF mAb in target retinal cells. Preclinical studies show promise of yielding high levels of protein expression compared to previous AAV vectors. Currently, phase II clinical trials are in progress to determine the tolerability of RGX-314 [182] when delivered via a subretinal injection during vitrectomy.

##### Other Factors

A concurrent expression of angiostatin and endostatin upon sub-retinal injection of EIAV-lentiviral particle (RetinoStat, NCT01301443) has been investigated in phase I [183]. Such a protein expression tool can lead to the production of both molecules from a single lentiviral vector. This methodology was first introduced by Lai et al. who demonstrated the effective inhibition of proliferative neovascularization in rat models upon intravitreal administration of AAV-endostatin or lentiviral vector-angiostatin [227]. The results from the phase I clinical trial are summarized in Table 5. Among the 21 test patients, three cases reported the development of a macular tear and hole in the retina. These patients were taken care of through further surgical interventions. Additionally, no specific complications were noted, and it was observed that high levels of the angiostatin and endostatin were maintained for a prolonged period. This indicates that further research in this genetic therapy-based therapeutic intervention is required. A recent study by Ling Sikai et al., reported a lentiviral system (mLP-CRISPR) prevented the development of wet AMD in a laser-induced CNV mouse model and did not induce Cas-9 specific immune responses after subretinal injections [184].

### 3.7. Cell Therapy

**Figure 4:**
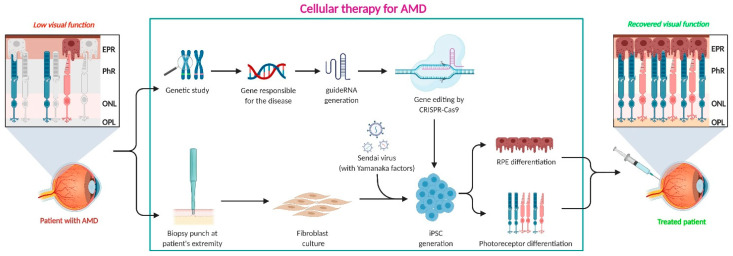
Cell therapy strategies for neovascular AMD.

Recent studies have suggested that the treatment modalities in clinical practice do not specifically target the degenerative nature implicated in the disease manifestation, thereby leading to possible disease recurrence and adverse effects in patients [185,228]. To overcome these challenges, stem cell therapy has been identified as a potential therapeutic strategy in the treatment of nAMD and possibly non-exudative AMD. Due to the capacity of stem cells to differentiate into various body tissues, this cell-based therapy fundamentally aims to reconstruct and restore the functionality of degenerated cells by cellular transplantation or stimulation of trophic factor secretion to maintain or restore visual acuity [7,8,9]. Cell therapy strategies for neovascular AMD are shown in Figure 4.

RPE cell transplantation employs either cell sheets with/without scaffolds or cell suspensions. There have been several previous publications that specifically identify devices for administering cell sheets for RPE transplantation [7,186,229,230]. In the case of administering cell suspension, a soft-tip subretinal cannula is used [231,232]. Studies suggest that the risk of complications arising due to the invasive nature of the surgical transplantation is high for sheets than suspension. This is primarily ascribed to the wide site of incision and possible CNV removal prior to the sheet transplantation [233,234].

Currently, induced pluripotent stem cell (iPSC) derived RPE cells, somatic stem cells (SSC), and embryonic stem cells (ESC) have been investigated for AMD therapy [185,228,235]. In 2013, Takahashi, in collaboration with RIKEN and Kobe City Medical Center, launched a clinical research project to evaluate the effect of autologous iPSC-derived RPE cell sheets upon transplantation [7]. At the five-year mark post-surgery, studies revealed that the relative stability of the sheet at the site of transplantation was satisfactory while preserving the pigmentation [229]. Further, the patients’ improved vision was retained at the five-year mark. The primary endpoints, including tumorigenesis, increased intraoperative pressure, failure, and rejection of transplantation were not observed. One of the notable results concluded that the corrected vision was maintained without the requirement of additional anti-VEGF therapy. At present, the National Eye Institute is evaluating the safety profile and feasibility of the subretinal transplantation of iPSC-derived RPE monolayer on PLGA scaffold as a prospective candidate for autologous cell therapy [236].

Following autologous stem cell therapy, allogeneic stem cell therapy was investigated. According to the reported results of the clinical trials, no significant intraoperative adverse effects were observed. During the transplantation procedure, anti-VEGF therapy and topical ocular steroids were administered to prevent rejection of the transplanted cells and treat the degenerated cells. No specific cases of rejection were reported. At the five-week mark after the surgery, the optical coherence tomography findings of one out of the 5 patients revealed mild immune rejection. In the end, all five patients with transplantation retained the grafted cells beyond 2 years, suggesting that the mild case of immune rejection can be positively managed with novel interventions [185].

Another phase I clinical trial (2015) with a sheet of allogeneic ESC-derived RPE cells was conducted for nAMD. Two nAMD patients underwent surgical transplantation of the RPE cell sheet employing ~10,000 ESC-derived RPE cells on vitronectin-coated polyester scaffold. According to the reported results in 2018, no specific adverse effect was observed within the one year following surgery, negating the direct cause–effect relationship between transplantation and post-surgical complications [9]. Unfortunately, studies did report that one patient suffered a retinal detachment and split lens sac immediately after transplantation. It was also noticed that no significant immune rejection occurred following administration of oral steroids [7,186,231]. Due to such breakthroughs in scaffold base therapy, bio-scaffolds composed of fibrin-based hydrogel or PLGA are being researched as advanced substitutes for RPE sheet transplantation [237,238].

SSCs (somatic stem cells) have also been researched in clinical trials to establish whether these improved or protected the trophic factor circulation from the transplanted cells rather than reconstruction of the retinal tissue. As per the recent clinical trials, most of the studies have employed bone marrow stem cells administered via the intrathecal route, subtenon sac, and retrobulbar injection. However, several cases of ocular hypertension, retinal and vitreous hemorrhage, retinal detachment, and dislocation of the lens has been reported after intravitreal transplantation of stem cells derived from the adipose tissue [239]. Such results encourage improvement in the assessment of efficacy evaluation method followed by the requisite alteration, optimization of resources for obtaining cell cultures, and innovation of novel technologies for cost-effective manufacturing processes.

## 4. Concluding Remarks and Future Perspectives

Over the past few decades, the irremediable blindness due to the fast progression of nAMD in the geriatric population has led researchers to shift their focus from intravitreal anti-VEGF biologics (Bevacizumab, Ranibizumab, Aflibercept, and Brolucizumab) to advanced therapeutic strategies that possess an improved safety and efficacy profile accompanied with minimal adverse effects. One step in this direction involves the exploration of therapy requiring administration of small molecules (Tyrosine Kinase Inhibitors and anti-inflammatory molecules), biologics, and gene products. In 2017, Luxturna® is approved for Leber’s congenital amaurosis (LCA) genetic disease, may boost further interest in gene therapy. Several research groups are focused on the progression of non-invasive delivery and sustained release strategies for the posterior segment. The Port Delivery System seems to be the front-runner in the long-acting strategy. Several particulate systems are under clinical and preclinical development. However, pre hand information of potency and dosage requirements are crucial in the success of such technologies. Most studies reported in this space are rather observational; quantitative estimations and mass balance of molecules kinetics is often missing. The therapeutic interest in radiation therapy is declining rapidly due to the emerging biologics. At present, bispecific antibodies are being continuously explored, and the first step towards their commercialization requires the reduction in the ocular cytotoxicity, followed by thorough investigation in clinical trials to expand the scope of therapy. BYOOVIZ™ (SB11), a biosimilar referencing LUCENTIS^®^ (Ranibizumab), recently approved by the U.S. Food and Drug Administration (FDA), may reduce the cost of treatment. Though anti-VEGF therapy has many challenges, it is going to remain the primary treatment until one of the described therapies effectively replaces it and subsequently improves patient compliance.

## Figures and Tables

**Table 1:** Molecular targeted strategies for nAMD treatment.

Molecular Target	Molecule Name	Specific Molecular Target	Format	Clinical Progress	Refs.
VEGF	Bevacizumab	All VEGF isoforms	Humanized full length monoclonal IgG1	Used clinically/Off-label	[40]
	Ranibizumab	All VEGF-A isoforms including VEGF 165, VEGF 121, and VEGF 110	Fragment of humanized monoclonal IgG1k	Used clinically	[44,45]
	Aflibercept	VEGF-A, B, C, and D isoforms	Fusion protein composed of binding domains of VEGFR-1 and 2 fused with the Fc region of IgG1	Used clinically	[46]
	Pegaptanib	VEGF 165 isoform	PEGylated form of a neutralizing RNA aptamer	Practically off the market or used in cases where the patient is already using	[47]
	Brolucizumab	All VEGF isoforms	scFv	Used clinically	[48]
	Abicipar pegal (MP0112)	All VEGF-A isoforms	PEGylated DARPin	Phase III clinical trials	[49]
	Conbercept	VEGF-A, B and C	Fc fusion	Phase III clinical trials with Lucentis^®^	[50]
	KSI—301	VEGF-A	IgG1 biopolymer conjugate	Phase II clinical trials	[51]
	OPT—302	VEGF-C and VEGF-D	Fc fusion	Phase III clinical trials	[52]
PDGF	Pegpleranib	PDGF-B	32-nucleotide PEGylated DNA aptamer	Phase III Clinical trials with Lucentis^®^	[53]
	Rinucumab	PDGF-B	IgG4	Phase II Clinical trials with Eylea^®^	[54]
ANG	Nesvacumab	ANG-2	IgG1	Discontinued Phase II Clinical trials with Eylea^®^	[43]
Bispecific targets	IBI302	VEGF/ Complement Activation System	Recombinant human anti-VEGF- and anti-complement bispecific fusion protein	Phase I Clinical trials	[55]
	RG7716	VEGF/ANG-2	Bispecific domain-exchanged mAb (CrossMAb)	Phase III Clinical trials	[56]
Small molecules and others	Triamcinolone acetonide	Multiple targets	Synthetic corticosteroid	Phase III Clinical trials with PDT	[57]
	Anecortave acetate	Protease	Glucocorticoid analogue	Phase II Clinical trials with PDT	[58]
	Sorafenib	VEGFR-1 and 2	TKI	Discontinued clinical trials	[59]
	Sunitinib Malate	TK receptors	TKI	Formulation Development	[60,61]
	Dexamethasone	Multiple targets	Synthetic glucocorticoid	Phase II clinical trials	[62]
	Levodopa	GPR143, PEDF, and VEGF	Amino acid precursor of dopamine	Phase II clinical trials	[63,64,65]

**Table 2:** Bispecific antibodies for AMD therapy [100].

Drug Name	Target	Sponsors	Phase	Status	ClinicalTrials.gov Identifier
Faricimab (RG7716)	VEGF-A, ANG-2	Hoffmann-La Roche	3	Active, Not recruiting/Enrolling by invitation/Recruiting	NCT03823287 NCT03823300 NCT04777201
			2	Completed	NCT03038880 NCT02484690
Faricimab (RO6867461)	VEGF-A, ANG-2	Chugai Pharmaceutical Co. Ltd.	1	Completed	JapicCTI-173634
IBI302	VEGF, CR1	Innovent Biologics Co. Ltd.	1	Active, Not yet recruiting	NCT03814291 NCT04370379
			2	Not yet recruiting	NCT04820452
BI 836880	VEGF-A, ANG-2	Boehringer Ingelheim	1	Recruiting	NCT03861234
RC28-E	VEGF, FGFR	RemeGen Co. Ltd.	1/2	Active, Not recruiting	NCT04270669
			1	Completed	NCT03777254

**Table 3:** Relationship between drug dose and dosing frequency for drugs with different target concentrations from pharmacokinetic simulations [123].

Type of Molecule	Vitreal Clearance	Target Concentration in Vitreous	Required Dose for 3 Months	Required Dose for 12 Months
Small molecule (500 Da)	0.05–1 mL/h	100 µM	30 mg	120 mg
		10 µM	3 mg	12 mg
		1 µM	305 µg	1.2 mg
		0.1 µM	30 µg	120 µg
Antibodies (149 k Da)	0.01–0.07 mL/h	10 µM	55 mg	220 mg
		1 µM	5.5 mg	22 mg
		0.1 µM	550 µg	2.2 mg
		10 nM	55 µg	220 µg

**Table 4:** Therapeutic and delivery strategies for nAMD.

Therapeutic Modality	Active Ingredient	Delivery System	Clinical Progress	Highlights	Refs.
Port Delivery System	Ranibizumab	Ocular implant	Clinical development	Sustained and controlled release of Ranibizumab to neutralize VEGF	[158]
Photodynamic Therapy	Verteporfin	Liposomes	Approved for clinical use (Visudyne^®^)	Cessation of bleeding and selective occlusion of the newly formed blood vessels along with decreased exudate formation	[165]
	Verteporfin	Cationic liposomes	Exploratory studies	CNV occlusion and reduced retinal deterioration compared to Visudyne^®^	[168]
	Paclitaxel/Succinyl-paclitaxel	Cationic liposomes	Exploratory studies	Inhibition of angiogenesis in rat models	[168]
	Hypocrellin B	Liposomes	Exploratory studies	Significant reduction in CNV area with reduced tissue damage	[169]
	Edaravone	Liposomes	Exploratory studies	Inhibition of ROS generation, reduced thickness of outer nuclear layer and no cytotoxicity	[170]
	Calcein	Gold nanoparticles	Exploratory studies	Light activated sustained and controlled drug release	[171]
	Calcein	ICG loaded liposomes	Exploratory studies	Light induced improved permeability of liposomes to control drug release	[172]
	Photocyanine and Sorafenib	RGD modified liposomes	Exploratory studies	Reduced CNV area and improved safety profile	[173]
	Verteporfin	Liposomes	Exploratory studies	Combination with anti-VEGF agents is more efficacious than anti-VEGF monotherapy	[174]
Gene therapy	PEDF gene	AAV	Clinical trials	Inhibition of angiogenesis	[175]
	PEDF gene	E1-, partial E3-, E4-deleted AAV	Clinical trials	No deleterious adverse effects were observed, however no concrete evidence is available to show the improved therapeutic efficacy and the relation between response and dose escalation	[176]
	sFLT-1	Recombinant AAV	Clinical trials	Antiangiogenic activity and reduced progression of CNV but no promising results	[177,178,179]
	ADVM-022 and ADVM-032	Vector capsid, AAV.7m8	Clinical trials	Antiangiogenic activity in murine models with laser induced CNV	[180]
	sFLT1	AAV2 with CBA promoter	Clinical trials	Higher expression with promoter introduction, however disappointing results	[181]
	Anti-VEGF mAb	AAV8 vector, RGX-314	Clinical trials	High protein expression therefore tolerability is being studied	[182]
	Angiostatin and Endostatin	EIAV-lentiviral particle	Clinical trials	No specific complications observed and high levels of active maintained.	[183]
	mLP-CRISPR	Lentiviral particle	Clinical trials	Prevented progression of nAMD and no specific immune responses	[184]
Stem cell therapy	Autologous iPSC derived RPE cells	Cell sheet	Clinical trials	Retained stability of transplanted sheet, corrected vision and no rejection of graft observed after 5 years	[7]
	Allogeneic iPSC derived RPE cells	Cell suspension	Clinical trials	No specific cases of graft rejection were observed and mild cases of immune rejection that can be managed	[185]
	Allogeneic ESC derived RPE cells	Cell sheet	Clinical trials	Few cases of immediate adverse effect observed, no immune rejection responses.	[186]

**Table 5:** Completed and ongoing gene therapy clinical trials for nAMD. Adapted from [182].

Expressed Gene	Vector	Phase	Route of Delivery	Status	Sponsor	Trial Registration Number
PEDF	AAV5	I	Intravitreal	Completed	GenVec	NCT00109499
sFLT01	AAV2	I/II	Subretinal	Completed	Lions Eye Institute, Adverum Biotechnologies	NCT01494805
Aflibercept	AAV2	I	Intravitreal	Ongoing	Adverum Biotechnologies	NCT03748784
sFLT01	AAV2	I	Intravitreal	Completed	Sanofi Genzyme	NCT01024998
Anti-VEGF Fab	AAV8	I/IIa	Subretinal	Ongoing	Regenxbio	NCT03066258
Endostatin and angiostatin	EIAV	I	Subretinal	Completed	Oxford BioMedica	NCT01301443
sCD59	AAV2	I	Intravitreal	Ongoing	Hemera Biosciences	NCT03585556

AV, adeno-associated viral vector; EIAV, equine infectious anemia lentiviral vector; and nAMD, neovascular age-related macular degeneration.

## Abbreviations

AMD	Age-related macular degeneration
AAV	Adeno associated virus
ANG	Angiopoietins
AREDS	Age-Related Eye Disease Study
ARM	Age-related maculopathy
AuNPs	Gold Nanoparticles
CNV	Choroidal neovascularization
DME	Diabetic macular edema
EBT	External beam therapy
EMBT	Epimacular brachytherapy
ESC	Embryonic stem cells
GA	Geographic atrophy
HSA	Human serum albumin
HUVECs	Human umbilical vein endothelial cells
ICG	Indocyanine green
IgG	Immunoglobulin G
iPSC	induced Pluripotent stem cell
mAb	Monoclonal antibody
NPs	Nanoparticles
PDGF	Platelet derived growth factor
PDS	Port delivery system
PDT	Photodynamic therapy
PEG	Polyethylene glycol
PGB	Pegaptanib
PLGA	Poly (D, L-lactide-co-glycolide)
PPV	Pars plana vitrectomy
ROS	Reactive oxygen species
RPE	Retinal pigment epithelium
SSC	Somatic stem cells
TKI	Tyrosine kinase inhibitor.
VEGF	Vascular endothelial growth factor
